# A Pilot Study of Peripheral Muscle Magnetic Stimulation as Add-on Treatment to Repetitive Transcranial Magnetic Stimulation in Chronic Tinnitus

**DOI:** 10.3389/fnins.2018.00068

**Published:** 2018-02-20

**Authors:** Veronika Vielsmeier, Martin Schecklmann, Winfried Schlee, Peter M. Kreuzer, Timm B. Poeppl, Rainer Rupprecht, Berthold Langguth, Astrid Lehner

**Affiliations:** ^1^Department of Otorhinolaryngology, University of Regensburg, Regensburg, Germany; ^2^Department of Psychiatry and Psychotherapy, University of Regensburg, Regensburg, Germany

**Keywords:** rPMS, rTMS, tinnitus, brain stimulation, muscle magnetic stimulation, chronic tinnitus

## Abstract

While brain stimulation techniques have been examined as treatment options for chronic tinnitus for many years, they have recently been extended to multimodal treatment approaches. As chronic tinnitus is often accompanied by comorbid muscular tension in the neck and back, we performed a one-arm pilot study to explore the feasibility of a new multimodal treatment approach. In detail, repetitive peripheral magnetic stimulation (rPMS) of the back was performed before and after each session of repetitive transcranial magnetic stimulation (rTMS) of the brain. Data of 41 patients were analyzed, all of which were treated with ten sessions of rTMS of the left prefrontal and left temporoparietal cortex followed by rPMS of the neck and back muscles. Tinnitus severity was measured using the tinnitus questionnaire (TQ). Neck pain was assessed using the neck pain and disability scale (NPAD). The new treatment approach was feasible and well accepted by the majority of patients. However, the overall patient group did not improve significantly in either of the questionnaires. If patients were divided in different subgroups depending on whether they were suffering from neck pain or somatosensory tinnitus, explorative *post-hoc* tests suggested differential effects: patients with both neck pain and somatosensory tinnitus had better outcomes than patients without those conditions or with neck pain only. This was true for both the TQ and the NPAD. This effect was of transient nature though: the TQ score went back to its baseline level after a follow-up period of 12 weeks. Based on our results we recommend that in studies that investigate tinnitus treatments targeting somatosensory afferents patients should be stratified according to somatic co-morbidities and somatosensory influence on the tinnitus percept.

Clinical trial registration: www.clinicaltrials.gov, NCT02306447.

## Introduction

Chronic subjective tinnitus is a very heterogeneous condition with respect to its causes, clinical characteristics and the emotional distress perceived by a patient. Therefore, it has been suggested that there exist various subtypes of tinnitus which might respond to different treatment approaches (Landgrebe et al., 2010). Accordingly, there are also multiple models for tinnitus pathophysiology, all of which might be able to explain different aspects of tinnitus generation or maintenance. While cognitive models highlight the importance of top-down mechanisms such as selective attention, interpretation and emotional evaluation of the phantom sound (McKenna et al., 2014; Elgoyhen et al., 2015; Ghodratitoostani et al., 2016), there are also pathophysiological models of tinnitus which emphasize bottom-up influences by suggesting neuroplastic changes in somatosensory afferents (Shore et al., 2016). The current study seeks to target both bottom-up and top-down mechanisms by using a combined treatment of rPMS (hypothesized bottom-up influence) and rTMS (hypothesized top-down influence via cortical stimulation of DLPFC and temporoparietal cortex).

Tinnitus has been shown to be accompanied by altered activity of and connectivity between different cortical networks including temporal, parietal and frontal cortices (Schlee et al., 2009; Schmidt et al., 2013; Elgoyhen et al., 2015). As rTMS is considered to be able to interfere with alterations of cortical activity, it has been examined as a treatment option for patients suffering from tinnitus (Theodoroff and Folmer, 2013; Lefaucheur et al., 2014). The effect sizes for this treatment remain small (Lefaucheur et al., 2014). Therefore, different strategies have been tried to increase treatment effects such as targeting multiple brain areas with rTMS (Kreuzer et al., 2011; Lehner et al., 2016) or varying the frequency by which the rTMS pulses are applied (Schecklmann et al., 2016). Up to now, the stimulation of temporal and frontal cortical areas has been suggested to exert beneficial effects on tinnitus (Kleinjung et al., 2008; Langguth and De Ridder, 2013).

Besides the importance of auditory and non-auditory cortical structures, there is also strong evidence for the somatosensory bottom-up system to be involved in tinnitus pathophysiology. Even if controversial, one tinnitus subtype might be cervicogenic somatic tinnitus (Bhatt et al., 2015; Michiels et al., 2015). It is known that auditory-somatosensory integration takes place in the cochlear nucleus (Dehmel et al., 2008) and auditory brainstem activity was shown to be modulated by trigeminal and also somatosensory stimulation (Dehmel et al., 2012; Markovitz et al., 2015). Somatosensory inputs are thought to be functionally relevant with respect to suppression of body-generated sounds (Shore and Zhou, 2006). Pathological conditions are supposed to spread into to auditory system via the cochlear nucleus. Actually, plastic changes in this bimodal system have already been observed in animal models of tinnitus (Dehmel et al., 2012). Furthermore, many patients suffering from tinnitus are able to modulate their phantom sound by moving face or neck muscles (Levine et al., 2007; Sanchez and Rocha, 2011). This somatosensory tinnitus component has already been targeted by different treatment approaches. For instance, myofascial trigger point deactivation was shown to bring tinnitus relief for patients with tinnitus and comorbid myofascial pain syndrome (Rocha and Sanchez, 2012). There is also some evidence that the reduction of muscle tension of neck and back muscles can bring relief to some tinnitus patients. For example, it was shown that Qigong—a system of movements, body postures and breathing exercises—leads to an improvement of tinnitus severity especially in patients with somatosensory tinnitus (Biesinger et al., 2010). Additionally, a recent case report describes a patient whose tinnitus disappeared after the application of a cervical collar, underscoring the involvement of cervical muscles in tinnitus generation (Bechter et al., 2016). In a very recent study, Marks et al. (2018) found that bimodal auditory-somatosensory treatment was effective in reducing tinnitus loudness and severity in patients suffering from somatic tinnitus. With respect to rTMS, it has been hypothesized that rTMS effects may also be partly mediated by modulation of somatosensory afferents (Vanneste et al., 2011; Lehner et al., 2012). There is some preliminary evidence that magnetic stimulation can also be used for reducing muscle tension in neck muscles and for inducing analgetic effects (Smania et al., 2003, 2005; Zunhammer et al., 2011; Sollmann et al., 2016).

Only recently, brain stimulation techniques have been extended to multimodal treatment approaches by combining them with e.g., acoustic stimulation (Shekhawat et al., 2015) or relaxation techniques (Kreuzer et al., 2016). Integrating the knowledge about the central nervous dysfunction as well as the importance of the somatosensory system for chronic tinnitus, we investigate a new multimodal treatment approach which targets both systems by combining rTMS with repetitive peripheral magnetic stimulation (rPMS) of the neck muscles. For rTMS, a stimulation protocol was chosen which combines low-frequency stimulation of auditory cortical areas with high-frequency stimulation of the prefrontal cortex and which has already shown promising effects in the past (Kleinjung et al., 2008; Langguth et al., 2014). While low-frequency rTMS of the temporoparietal cortex is a standard procedure (Lefaucheur et al., 2014) high frequency rTMS of the prefrontal cortex is supposed to induce activity changes in the anterior cingulate cortex (Speer et al., 2000) which is thought to be involved in tinnitus distress (Vanneste et al., 2010). rPMS treatment is supposed to bring relief to muscle tension (Smania et al., 2003, 2005) which might alter the somatosensory input to the cochlear nucleus. We investigated the feasibility of this bimodal treatment approach in a one-arm pilot study (Dobie, 1999; Landgrebe et al., 2012).

## Materials and methods

### Subjects

The study was registered at Clinical Trials (NCT02306447). Inclusion criteria for study participation were age between 18 and 80 years and presence of chronic subjective tinnitus for at least 6 months. Exclusion criteria were objective tinnitus, a treatable cause of tinnitus and the involvement in other treatments for tinnitus at the same time. Furthermore, patients with clinically relevant psychiatric comorbidities, alcohol or drug abuse, acute neck or back pain, neck or back pain with unknown etiology as well as unstable internal or neurological comorbidities were excluded. In addition, general exclusion criteria for rTMS or rPMS stimulation applied (history or evidence of significant brain malformation or neoplasm, head injury, cerebral vascular events, neurodegenerative disorders affecting the brain, prior brain surgery, metal objects in and around the body that cannot be removed, pregnancy). Patients were recruited during routine clinical tinnitus consultations. All data were collected at the Department of Psychiatry and Psychotherapy, University of Regensburg between September 2014 and April 2016 (last follow-up visit). All research participants provided written, informed consent to participate in this research as well as for the data to be used for analysis and publication. Data were gathered and analyzed within the framework of the Tinnitus Research Initiative database (Landgrebe et al., 2010) which was approved by the Ethics Committee of the University Hospital of Regensburg (Germany, reference number 08/046).

### Questionnaires and outcome measures

Patients completed the below listed questionnaires at four measurement time points: at baseline (treatment day 1), week 2 (treatment day 10, last treatment day), week 4 and week 12 (2 and 10 weeks after the last treatment session, respectively). Tinnitus severity was assessed using the German version of the Tinnitus Questionnaire (TQ, Goebel and Hiller, 1994), the Tinnitus Handicap Inventory (THI, Newman et al., 1996) and five rating scales measuring how loud, uncomfortable, annoying, unpleasant and how easy to ignore the tinnitus was. Those scales ranged from 0 (not at all loud/uncomfortable etc.) to 10 (extremely loud/uncomfortable etc.). In addition, depressive symptoms were assessed by the Major Depression Inventory (MDI) and quality of life was measured by the WHO-QoL BREF (World Health Organization Quality of Life) assessment which is divided into four domains: physical health (domain 1), psychological health (domain 2), social relationships (domain 3), and environment (domain 4). In addition, patients completed the neck pain and disability scale (NPAD, Scherer et al., 2008) at baseline and week 2. The NPAD was only available for a subgroup of 34 patients though. In order to assess demographic and clinical patient characteristics at baseline, patients filled in the Tinnitus Sample Case History Questionnaire (Langguth et al., 2007) and underwent pure-tone audiometry. The mean hearing threshold is reported which represents the average of all thresholds measured bilaterally for frequencies between 125 Hz and 8 kHz.

Primary outcome was defined as the change of tinnitus severity as measured by the TQ from baseline to week 12. Secondary outcomes were changes in TQ, THI, MDI, numeric rating scales, and WHO-QoL over the course of the trial (baseline, week 2, week 4, and week 12). Furthermore the change in the neck pain and disability scale (NPAD) from baseline to week 2 was analyzed.

### rTMS and rPMS treatment

**Figure 1:**
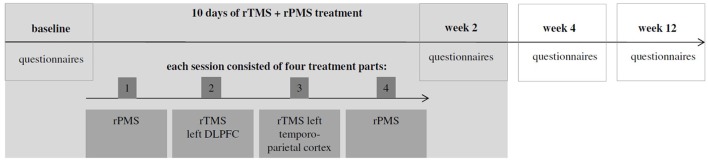
Overall treatment schedule.

The present clinical trial was designed as a one-arm open-label proof of concept study. Therefore, all patients underwent the same treatment procedures during which they were treated in 10 sessions on 10 consecutive working days with a break over the weekend. Each treatment session consisted of four parts which were applied successively without break in between (apart from the break which was necessary to change coils; see Figure 1): (1) rPMS of the neck and back muscles; (2) rTMS stimulation of the left dorso-lateral prefrontal cortex (DLPFC, 2000 stimuli, 20 Hz, which were applied in 20 trains with an intertrain interval of 25 s); (3) rTMS stimulation of the left temporo-parietal cortex (2000 stimuli, 1 Hz). (4) rPMS of the neck and back muscles.

**Figure 2:**
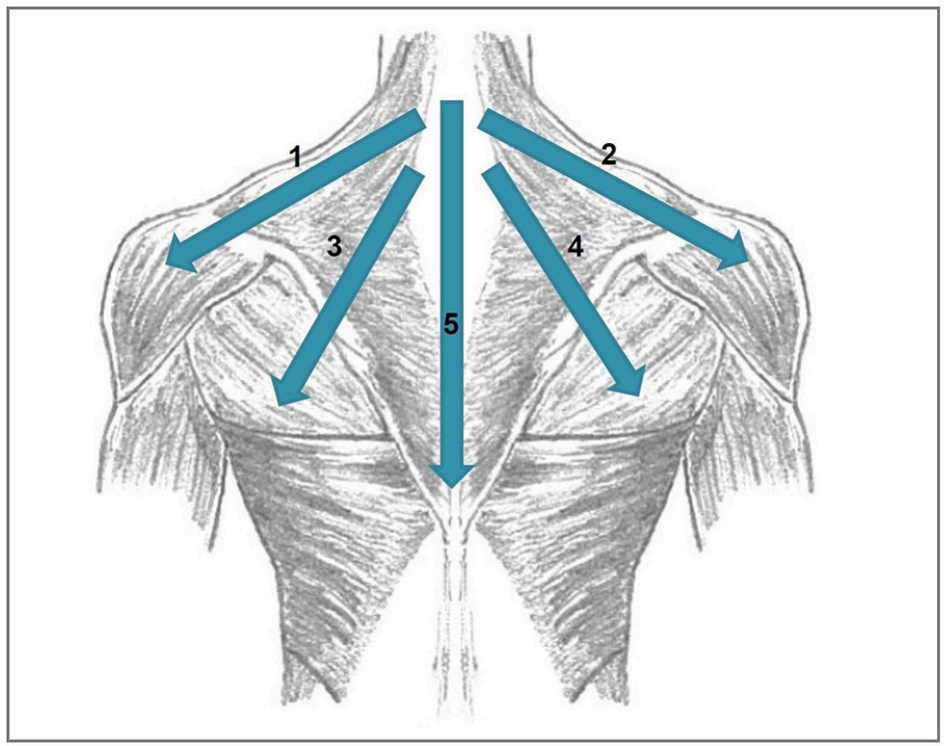
Directions of the movements done during rPMS stimulation. 1, left trapezius and deltoid muscle; 2, right trapezius and deltoid muscle; 3, left trapezius and latissimus dorsi muscle, 4, right trapezius and latissimus dorsi muscle; 5, cranio-caudal movement over the backbone.

(2) and (3) were done at a stimulation intensity of 110% resting motor threshold using a Medtronic MagPro X100 stimulator (Medtronic, Denmark) and a 70 mm figure-of-eight coil. The temporo-parietal cortex was localized using the 10–20 system: The coil was placed between the temporal (T3) and parietal (P3) EEG electrode sites. The DLFPC was targeted by centring the TMS coil 6 cm anterior from the part of the motor cortex which had been used for defining the motor threshold (Lehner et al., 2013). Combined temporoparietal plus frontal stimulation protocol have been examined before and were shown to be safe (Langguth et al., 2014; Kreuzer et al., 2015; Lehner et al., 2016). The rPMS protocol was based on clinical experience in the use of rPMS in rehabilitative medicine and consisted of four medial-lateral movements starting from the neck (1: left trapezius and deltoid muscle; 2: right trapezius and deltoid muscle; 3: left trapezius and latissimus dorsi muscle; 4: right trapezius and latissimus dorsi muscle) and one cranio-caudal movement over the backbone (see Figure 2). The series of those five movements was repeated eight times: the first four repetitions with a stimulation frequency of 5 Hz, the remaining four repetitions with 20 Hz. Each movement consisted of 20 stimulation pulses. As a consequence, the duration of a 20 Hz movement was 1 s, the duration of a 5 Hz movement was 4 s. Between the movements, there was a 2s interval. In total, one rPMS treatment part had a duration of 100 s. rTMS treatment lasted 2,575 s (2,000 s for the 1 Hz treatment, 575 s for the 20 Hz treatment incl. intertrain intervals). As a consequence, a complete session of two rPMS treatment parts plus rTMS treatment lasted 2,775 s or 46.25 min. rPMS stimulation was done using a round coil with 126 mm outer diameter (MagVenture MMC-140-II) at an intensity that was determined as individually comfortable in a pretest (typically 20–30% of maximal stimulator output). Before the first treatment session, the resting motor threshold was measured. It was defined as the minimal intensity at which at least five of ten motor evoked potentials were 50 μV in amplitude in the right abductor digiti minimi.

### Statistical analysis

For statistical analyses IBM SPSS Statistics for Windows (Version 22.0, Armonk, NY: IBM Corp.) was used. Four missing values were replaced by using a last observation carried forward (LOCF) procedure: The TQ score of one patient was missing for the final visit and the score for the rating scale “annoying” was missing for another patient for week 2. Furthermore, the MDI score for week 2 was missing for one patient and for week 12 for another patient. Recently, we could demonstrate that the LOCF method induces no statistical bias in comparison to linear mixed effects analyses for missing data <10% (Kreuzer et al., 2016). The changes of the TQ score from baseline to week 12 (primary outcome) and of the NPAD score from baseline to week 2 were tested using paired *t*-tests with the within-subjects factor measurement time point. To test for changes in tinnitus severity scores, MDI and WHO-QoL over all four measurement time points an analysis of variance (ANOVA) with the within-subjects factor measurement time point (baseline, week 2, week 4, week 12) was calculated for all questionnaires and rating scales. For the ANOVAs, the sphericity of data was checked with Mauchly Tests (Mauchly, 1940). In case of significant Mauchly-Tests, Greenhouse-Geisser corrections were applied.

In addition to the statistical analyses described above, some exploratory data analyses were conducted in order to understand the results in more detail. To this end, some analyses with a special focus on neck pain and somatosensory tinnitus were done. Patients were divided into different groups depending on whether they were suffering from neck pain (“Do you suffer from neck pain?”) and/or from somatosensory tinnitus (“Does any head and neck movement (e.g., moving the jaw forward or clenching the teeth), or having your arms/hands or head touched, affect your tinnitus?”), based on their answers in the Tinnitus Sample Case History Questionnaire (Langguth et al., 2007). A number of 11 patients did not suffer from neck pain or somatosensory tinnitus, 16 patients suffered from neck pain only, and 11 patients reported both neck pain and somatosensory tinnitus. Another 3 patients only reported somatosensory tinnitus. Because of the small sample size, this group was excluded from the following analyses. For the subgroup of 34 patients who filled in the NDPAD, 9 patients did not suffer from neck pain or somatosensory tinnitus, 13 suffered from neck pain only, 9 reported both neck pain and somatosensory tinnitus and 3 reported somatosensory tinnitus only.

Repeated measures ANOVAs were done to compare the resulting three groups with respect to the change of the NPAD score and the TQ from baseline to week 2. The homogeneity of variances between groups was tested with Levene's Tests. In case of significant Levene's Tests, F_max_-Tests were done. Those tests revealed that an adaptation of the level of significance was not necessary for the ANOVAs with the TQ as dependent variable. For the ANOVA with the NPAD as dependent variable, the significance level had to be adapted to.025.

## Results

### Dropouts

Forty-nine patients were enrolled in the study. Three patients dropped out of the study during the treatment phase. One of them reported a light subjective cardiac arrhythmia. Although he had had cardiac arrhythmias before and the relation to rPMS seemed to be doubtful, the rPMS treatment was terminated. Another patient dropped out due to an ongoing loudening of the tinnitus percept. The third patient dropped out due to a hypertensive crisis with doubtful relation to rTMS treatment (pre-known hypertension). Five further patients dropped out of the study after the treatment phase during the follow-up phase, all for unknown reasons. One of them had described a transient loudening of the tinnitus before. All in all, data of 41 patients were left to be statistically analyzed (see Table 1 for demographic and clinical characteristics of this sample at baseline).

**Table 1:** Demographical data and clinical characteristics at baseline (M ± SD) for the overall patient group and for the three exploratory subgroups.

	**Overall patient group (*n* = 41)**	**Neither neck pain nor somatosensory tinnitus (*n* = 11)**	**Neck pain (*n* = 16)**	**Neck pain and somatosensory tinnitus (*n* = 11)**
Age (years)	50.70 ± 12.69	48.21 ± 12.67	52.20 ± 11.67	53.62 ± 14.37
Gender	26 m, 15 f	8m, 3f	9m, 7f	6m, 5f
Mean hearing threshold	18.18 ± 11.84	15.01 ± 10.22	21.59 ± 10.89	20.45 ± 13.26
[dB HL]	(*n* = 40)		(*n* = 15)	
Tinnitus laterality (r/l/l>r/r>l/both/inside head)	6/10/8/6/8/3	0/5/2/0/4/0	2/5/3/1/4/1	3/0/3/4/0/1
Tinnitus duration in years	7.69 ± 7.70	11.07 ± 8.80	4.34 ± 4.93	8.95 ± 9.11
	(*n* = 38)	(*n* = 10)	(*n* = 15)	(*n* = 10)
TQ (0–84)	37.83 ± 16.23	25.27 ± 16.62	41.19 ± 16.85	43.27 ± 8.01
THI (0–100)	42.34 ± 21.57	31.73 ± 21.99	44.63 ± 22.71	48.36 ± 16.46
MDI (*N* = 40; 0–50)	7.23 ± 5.43	4.36 ± 4.91	8.44 ± 6.11	8.55 ± 4.59
WHO-QoL Domain 1 (4–20)	15.47 ± 2.57	17.12 ± 2.37	14.52 ± 2.87	15.21 ± 1.77
WHO-QoL Domain 2 (4–20)	14.35 ± 2.66	15.12 ± 3.43	13.98 ± 2.17	14.10 ± 2.90
WHO-QoL Domain 3 (4–20)	15.62 ± 2.85	15.76 ± 3.15	16.10 ± 2.63	15.27 ± 3.00
WHO-QoL Domain 4 (4–20)	16.71 ± 1.62	17.91 ± 1.76	16.16 ± 1.21	16.77 ± 1.49
Rating scales (0–10)				
Strong/loud	6.73 ± 1.88	5.55 ± 2.16	7.13 ± 1.86	7.27 ± 1.49
Uncomfortable	6.76 ± 2.05	5.73 ± 2.01	6.75 ± 2.27	7.55 ± 1.64
Annoying	6.56 ± 2.18	5.27 ± 2.20	7.13 ± 2.28	7.00 ± 1.95
Ignoring	6.20 ± 2.52	4.45 ± 2.30	7.19 ± 2.54	6.55 ± 2.30
Unpleasant	6.68 ± 2.15	5.45 ± 1.92	6.87 ± 2.28	7.45 ± 2.12
Somatosensory tinnitus	14 yes, 27 no			
Suffer from neck pain	27 yes, 14 no			
NPAD score (0–100)	31.47 ± 24.26	4.22 ± 5.97	42.15 ± 19.04	47.33 ± 20.97
	(*n* = 34)	(*n* = 8)	(*n* = 13)	(*n* = 9)

*Mean hearing threshold (in dB HL): average of all thresholds measured bilaterally ranging from 125 Hz to 8 kHz. Tinnitus laterality is defined in categories: r, right-sided; l, left-sided, l > r, both sides but louder on the left side; r > l, both sides but louder on the right side; both, both sides; inside head, tinnitus is perceived in the middle of/ inside the head. TQ, Tinnitus Questionnaire; THI, Tinnitus Handicap Inventory; MDI, Major Depression Inventory; rating scales ranging from 0 (not at all loud/uncomfortable etc.) to 10 (extremely loud/uncomfortable etc.); WHO-QoL, World Health Organization-Quality of Life; NPAD, neck pain and disability scale*.

### Adverse events

In all treated patients, both the rPMS as well as the rTMS part of the treatment were tolerated without severe side effects. Among the 41 patients who completed the study 5 patients (13%) reported transient headaches and one patient (2.5%) reported headache which was still present at week 12. Furthermore, six patients (14.6%) complained of an increase in tinnitus loudness. In two of them, this increase was still present at week 12. Additionally, one patient reported a transient pain in his fingers.

### Statistical analysis

**Figure 3:**
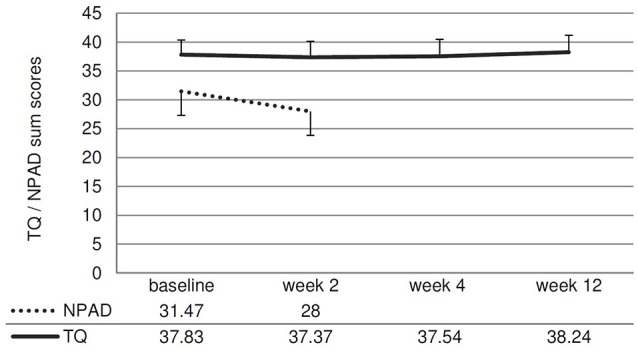
Line chart showing the NPAD and TQ scores over time. Error bars represent standard errors. The change of the NPAD sum score is marginally significant.

Concerning the primary outcome (change of the TQ score from baseline to week 12), no significant treatment effect was observed [*t*_(40)_ = −0.27; *p* = 0.787; *d* = 0.04]. The ANOVAs testing for changes in the different questionnaire scores and rating scales over all measurement time points were not significant (see Figure 3, Table 2). The NPAD score changed marginally from an average total score of 31.47 points at baseline to 28.00 at week 2 [*t*_(33)_ = 1.80; *p* = 0.081; *d* = 0.31).

**Table 2:** Results from repeated measures analyses of variance.

	***F* (*df*)**	***p***	***Eta^2^***
TQ	*F*_(3, 120)_ = 0.18	0.912	0.004
THI	*F*_(2.28, 91.23)_ = 0.27	0.792	0.007
MDI	*F*_(1.85, 72.09)_ = 0.90	0.404	0.023
Loudness	*F*_(3, 120)_ = 0.49	0.687	0.012
Uncomfortable	*F*_(3, 120)_ = 0.11	0.954	0.003
Annoyance	*F*_(3, 120)_ = 0.36	0.779	0.009
Ignoring	*F*_(3, 120)_ = 0.11	0.952	0.003
Unpleasant	*F*_(3, 120)_ = 1.11	0.348	0.027
WHO-QoL domain 1	*F*_(2.55, 102)_ = 1.40	0.250	0.034
WHO-QoL domain 2	*F*_(2.1, 83.88)_ = 0.22	0.810	0.006
WHO-QoL domain 3	*F*_(2.57, 102.78)_ = 1.20	0.312	0.029
WHO-QoL domain 4	*F*_(2.52, 100.74)_ = 1.90	0.144	0.045

### Exploratory data analysis

**Figure 4:**
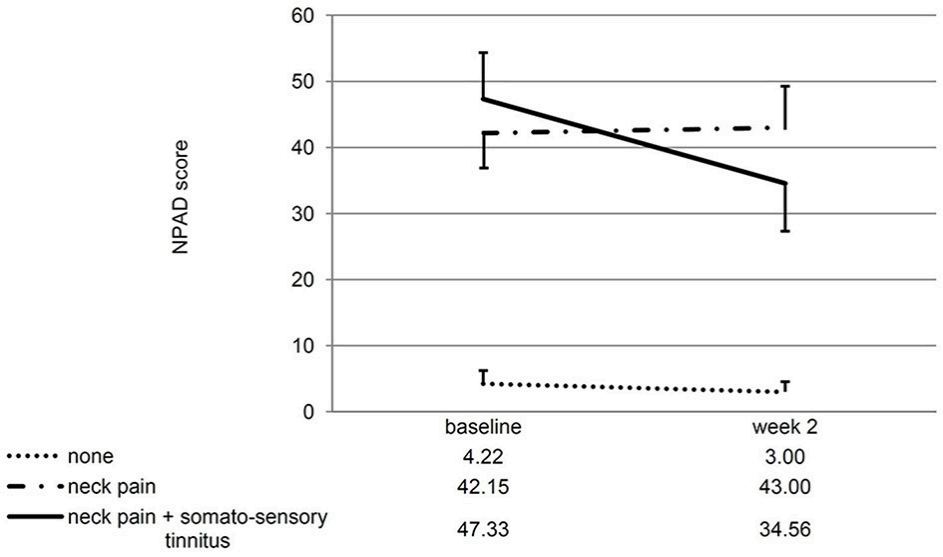
Line chart showing the NPAD score at baseline and week 2 for all three subgroups of patients. Error bars represent standard errors. The NPAD change of patients with both somatosensory tinnitus and neck pain differed significantly from the NPAD change of patients with neither condition. The difference to the change of patients with neck pain only was marginally significant.

**Figure 5:**
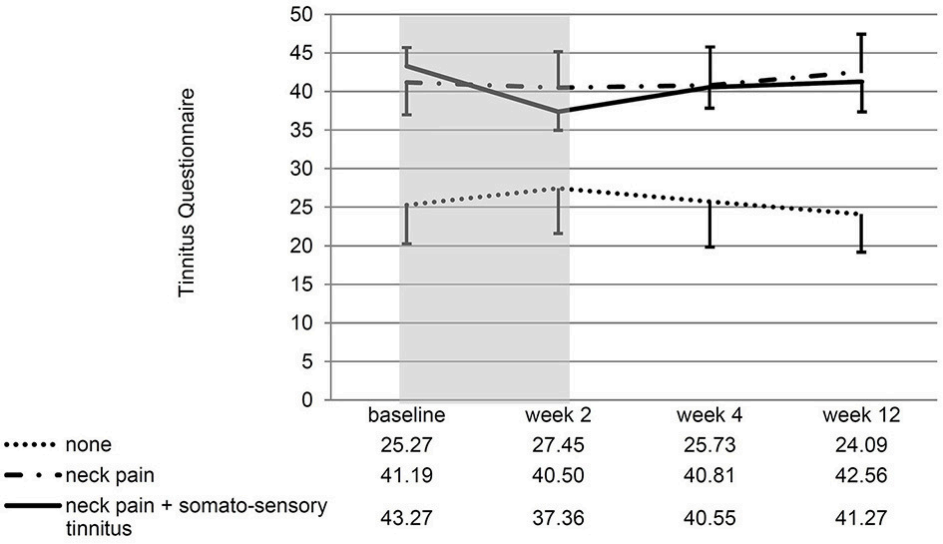
Line chart showing the score at all measurement time points for all three subgroups of patients. Error bars represent standard errors. The 10-day treatment phase is marked in gray color. The TQ change from baseline to week 2 of patients with both somatosensory tinnitus and neck pain differed significantly from the TQ change of patients with neither condition.

If the patients with/without neck pain and/ or somatosensory tinnitus were compared, the interaction effect time^*^group was significant for the change of the NPAD score from baseline to week 2 [*F*_(2, 28)_ = 4.88; *p* = 0.015; *eta*^2^ = 0.258]. For the three *post hoc t*-tests, the Bonferroni-corrected significance level has to be set at 0.016. *Post hoc t*-tests of the mean NPAD differences from baseline to week 2 revealed that patients with both neck pain and somatosensory tinnitus showed more NPAD change (*M* = −12.78; *SD* = 10.63) than patients with neck pain only (*M* = 0.85; *SD* = 13.20). This difference was marginally significant [*t*_(20)_ = −2.57; *p* = 0.018; *d* = 1.14]. Patients with both conditions also showed significantly more NPAD change than patients with neither condition [*M* = −1.22; *SD* = 2.77; *t*_(9.09)_ = −3.16; *p* = 0.011; *d* = 1.49]. There was no significant difference between the group with neither condition and the group with neck pain only [*t*_(13.50)_ = 0.55; *p* = 0.593; *d* = 0.22]. See Figure 4 for an illustration of the NPAD changes in all three groups. Also, the overall group effect was significant [*F*_(2, 28)_ = 15.73, *p* < 0.001; *eta*^2^ = 0.022]: patients without neck pain or somatosensory tinnitus scored lower on the NPAD than the other two patient groups. If the change of the TQ score from baseline to week 2 was analyzed, there was also a significant time^*^group interaction effect [*F*_(2, 35)_ = 5.47; *p* = 0.009; *eta*^2^ = 0.238]. Again, *post-hoc t*-tests of the mean TQ differences from baseline to week 2 (Bonferroni-corrected alpha = 0.016) revealed that the group with both conditions (*M* = −5.91; *SD* = 6.64) was significantly different from the group suffering from neither condition [*M* = 2.18; *SD* = 5.53; *t*_(20)_ = −3.11; *p* = 0.006; *d* = 1.32] and different by trend from the group suffering from neck pain only [*M* = −0.69; *SD* = 5.46; *t*_(25)_ = −2.24; *p* = 0.034; *d* = 0.86]. There was no significant difference between the group with neither condition and the group with neck pain only [*t*_(25)_ = −1.34; *p* = 0.194; *d* = 0.52]. Again, the main effect “group” was significant [*F*_(2, 35)_ = 3.35; *p* = 0.047; *eta*^2^ = 0.028]: patients without neck pain or somatosensory tinnitus scored lower on the TQ than the other two patient groups. If the TQ changes of all three subgroups were compared over all four measurement time points, the ANOVA revealed no significant time^*^group interaction effect [*F*_(4.46, 78)_ = 1.40; *p* = 0.238; *eta*2 = 0.074]. There was no significant main effect of time [*F*_(2.23, 78)_ = 0.44; *p* = 666; *eta*2 = 0.012] but a significant main effect of group [*F*_(2, 35)_ = 3.75; *p* = 0.033; *eta*2 = 0.177]. See Figure 5 for an illustration of the TQ changes in all three groups.

## Discussion

This is the first study to report combined rTMS and rPMS for the treatment of patients suffering from chronic subjective tinnitus. As it was designed as a pilot study, there are some weaknesses which should be kept in mind when interpreting the results. First, in order to examine the feasibility of the combined treatment, a one-arm trial with no control group was chosen. Second, we chose a well-studied standard rTMS protocol in order to combine it with rPMS treatment. This standard rTMS protocol consisted of left-hemispherical treatment and did not account for tinnitus laterality. The question if and how tinnitus laterality should be considered for the choice of the “right” rTMS treatment protocol has partly been examined for unilateral tinnitus (Khedr et al., 2010) but is still an open question for bilateral tinnitus or tinnitus which is perceived inside the head. A recent study indicates that tinnitus laterality has no association with rTMS response (Lehner et al., 2012). Although important, it was not part of the current study's hypothesis to add to this question. Third, the exploratory analyses were done post-hoc, which means that also the subdivision of patients to the different subgroups was done post-hoc. Therefore, the subgroups were not matched with respect to demographical and clinical characteristics.

It was shown that this treatment approach is feasible in clinical routine. The amount of side effects in the current study is similar to previous results. For example, in Lehner et al. (2016), 12% of the patients treated with triple-site stimulation and 25% of patients treated with standard single-site left temporoparietal stimulation reported transient headaches. In Kreuzer et al. (2015) 16.7% of patients reported headaches after combined left temporoparietal plus left DLPFC stimulation. In the current study, 13% of the patients reported transient, 2.5% ongoing headaches. An increase in tinnitus loudness was reported in 12.5% of the patients treated with single-site stimulation in Lehner et al. (2016), 11.1% in Kreuzer et al. (2015) and in 14.6% of the patients in the current study.

However, there was no significant improvement in tinnitus severity (as measured by the TQ) or neck pain (as measured using the NPAD). This outcome is worse than results of previous studies examining combined frontal plus temporal rTMS stimulation protocols (e.g., Kleinjung et al., 2008; Langguth et al., 2014). One promising approach in increasing treatment effects in chronic tinnitus is the combination of different therapeutic approaches. In tinnitus, several multimodal approaches including rTMS combined with relaxation (Kreuzer et al., 2016), transcranial electric stimulation combined with hearings aids (Shekhawat et al., 2014), tinnitus retraining therapy (Rabau et al., 2015) or tailor-made notched music therapy (Teismann et al., 2014; Lee et al., 2017), vagal nerve stimulation paired with acoustic stimulation (Li et al., 2015) or trigeminal nerve stimulation combined with acoustic stimulation (Hamilton et al., 2016) were introduced. Beside tinnitus other neuropsychiatric disorders were also the focus of combined therapies—for example combined brain stimulation and cognitive training in dementia (Nguyen et al., 2017) or combined brain stimulation and physiotherapy after stroke (Elsner et al., 2017; Salazar et al., 2018). Combination of different neuronal treatments is a challenging task as several open issues have to be resolved. The most important one is the timing or temporal order of both therapies (Bajbouj and Padberg, 2014; Martin et al., 2014; Marks et al., 2018). Combining different therapies might not only result in augmentation of effects. Complex interaction effects might also lead to reduced efficacy. The present trial combined rTMS with preceding and succeeding rPMS. This was a pilot study showing the feasibility and efficacy of the combined approach. In this study we found that for the whole group the combined approach had no beneficial effects, neither on neck muscle pain, nor on tinnitus severity. A possible explanation for this result is that the combination of cortical rTMS with rPMS, as it was investigated in this trial, is not synergistic in the overall patient group. Furthermore this combined protocol may act differentially on different subgroups of patients. This is in line with the findings of the exploratory analyses which suggest additive effects, i.e., linear increase of efficacy from the group without additional conditions over the group with neck pain to the group with neck pain and somatosensory tinnitus. Another explanation of course is that TMS is not effective in tinnitus. A recent review article concluded that it is possibly effective (Lefaucheur et al., 2014).

Nonetheless, a reduction of the TQ score of nearly 6 points from baseline to week 2 is rather large as compared to other rTMS studies. For example, Langguth et al. (2014) reported TQ changes of 2 points from baseline to week 2 for left temporal stimulation and of 3.32 points for a combined left temporal plus frontal stimulation. Lehner et al. (2016) reported a difference of 4.59 points in the TQ score from baseline to week 2 for the overall patient group. Up to now, the most effective treatment option for patients suffering from chronic tinnitus is a specialized care treatment protocol as suggested by Cima et al. (2012) where TQ differences of 7.38 points are seen after 3 months and 15.96 points after 12 months. On an individual patient level a reduction of 5 points in the TQ has been identified a minimal clinically important difference (Adamchic et al., 2012). If related to these results, a mean reduction by 6 points seems to be a rather large change which might be worth future research.

Importantly, the subgroups also differed with respect to the treatment outcome concerning neck pain: patients with both neck pain and somatosensory tinnitus improved with respect to the NPAD score while patients with neck pain only did not. All in all, this suggests that tinnitus patients with both conditions might represent a subgroup of patients for which combined rPMS and rTMS might be a promising treatment approach. There are different possible explanations for these findings. There are studies backing the hypothesis that an improvement of muscle tension leads to an improvement of tinnitus severity (Biesinger et al., 2010; Bechter et al., 2016). A recent systematic review has shown that cervical physical therapy is an effective treatment approach for patients with somatosensory tinnitus (Michiels et al., 2016). Furthermore, Marks et al. (2018) reported that a combined auditory-somatosensory treatment was able to reduce tinnitus loudness and severity in patients suffering from somatosensory tinnitus whereas unimodal auditory treatment was not, emphasizing the importance of the somatosensory system in these patients. This bimodal stimulation examined by Marks et al. has been shown to exert its effects via long term depression in the cochlear nucleus. The changes we observed in patients with neck pain and somatosensory tinnitus in our study might be mediated by similar mechanisms. The fact that we observed improvement only in patients with neck pain and somatosensory tinnitus suggests that both altered neuronal input from the neck area and an interaction between the somatosensory system and the tinnitus percept represent a requirement for a beneficial effect of rPMS.

This result emphasizes the relevance of individualized treatment for tinnitus patients. Tinnitus should be understood as a symptom with diverse causes and variable subgroups all of which might benefit from different treatment approaches (Landgrebe et al., 2010). Besides somatic tinnitus (Ward et al., 2015), typewriter tinnitus was defined as a very specific subtype which is responsive to carbamazepine (Levine, 2006). Further subtypes such as trauma-associated tinnitus (Kreuzer et al., 2012) or tinnitus in combination with specific comorbid symptoms such as temporomandibular joint disorders (Vielsmeier et al., 2011) have been reported. Therefore, bottom-up oriented treatment strategies might be useful for a different group of tinnitus patients than top-down oriented treatment options.

For electromagnetic stimulation, individual differences in the response to different central and peripheral stimulation techniques have already been demonstrated in the past (Vanneste et al., 2011). As brain stimulation effects depend particularly on the excitability state of the stimulated structure (Rossini et al., 2015), individualized treatment might be particularly relevant for treatment with electrical or magnetic stimulation. Moreover the combination of two techniques—such as rPMS plus rTMS—is challenging to explore, as the complexity is increased by additional aspects such as the temporal relationship between peripheral and central stimulation. Consequently, future studies should try to concentrate on subgroup-specific effects of different treatment strategies or, more generally, on individualized treatment programs considering the very specific combination of possible causes and/or tinnitus-related alterations of a particular patient (Kreuzer et al., 2017).

## Author contributions

BL and MS conceived the idea of the study. VV, PK, and TP contributed to data acquisition. AL analyzed the data and drafted the manuscript. All authors contributed to the interpretation of the result and revised the manuscript. All authors approved its final version. The authors declare no competing financial interests with respect to the study.

### Conflict of interest statement

The authors declare that the research was conducted in the absence of any commercial or financial relationships that could be construed as a potential conflict of interest.
